# "A Christmas Dream" at Birmingham

**Published:** 1914-01-03

**Authors:** 


### "A Christmas Dream" at Birmingham.

IN spite of the fact that the General Hospital, Birmingham, had been overfull, several wards having "extra" beds up, the preparations for Christmas were carried out with most pleasing results. On Christmas Eve the night nurses visited all the wards, before going on duty, with a portable harmonium and sang a number of carols. On Christmas morning each patient found a parcel containing some useful article of clothing or other gift on his or her locker, while the children, of whom there were eighty, found stockings of goodly dimensions quite full of toys and games. Holy Communion was celebrated in the chapel at 6.15 and 8 A.M., while many patients participated in the various wards who were unable to go to the chapel—in all there were 101 communicants. At 9.30 the Lord Mayor and Lady Mayoress, Lieutenant-Colonel and Mrs. Martineau, visited the hospital, the former being conducted round the male wards by the house governor, Mr. Howard Collins, and the Lady Mayoress visiting the female wards with the matron, Miss Musson. At 11 A.M. the chapel was filled with officers, nurses, servants, and patients. Sir John Holder, chairman of the house committee, also attended. The Bishop of Birmingham read the Lessons and preached on the subject of "Peace." The Bishop and Sir John Holder afterwards visited the wards, accompanied by the matron, the resident surgical officer, Mr. Sampson, and the house governor and Mrs. Collins. The wards were tastefully decorated with lamp-shades, etc., each ward having its own colour scheme, which had been kept a profound secret by sister and her nurses. Christmas dinner was served at 12.30, and consisted of roast beef and plum-pudding, or chicken and custard for the less robust—the carving being undertaken by the resident officers, many attired as *chefs*. The visitors to patients were allowed an extra half-hour, and each patient had two visitors to tea in the wards, a privilege that was much appreciated. The day closed with a ward concert held in one of the accident wards, the performers being residents and nurses. The night nurses had their Christmas dinner at 9.15 A.M., the day nurses at 1.15, and the matron and sisters at 7 P.M.

On Boxing Day the male patients were supplied with pipes and tobacco or cigarettes, and were allowed the great treat of smoking in the wards during the afternoon. On Boxing Day also the servants kept their Christmas, dinner being served at 2 P.M., and were waited on by the residents in weird costumes, the matron and house governor carving up three large turkeys and several puddings. In the evening the out-patient hall, which had been tastefully decorated by the out-patient sister and nurses, was transformed into a theatre. A stage, with electric foot and fly lights and scenery, was erected at one end. The end consulting rooms were utilised as green room and dressing rooms. The residents performed a duologue, "Train de Luxe," and the play "Vice Versa" most creditably—the sight of H.P.s and H.S. as school-boys in Eton coats and collars causing some of the committee and visiting staff considerable surprise. The "ladies'" parts were well done, and the leading part of "Dick" showed exceptional histrionic power.

On Saturday night the nursing staff gave their performance, over 140 patients being again taken to the out-patient hall on stretchers, couches, and wheel-chairs, and others accommodated on forms. This was an original play of a "Christmas Dream." The prologue showed a "child" (the tallest sister) in a cot in her nursery with toys and being put to sleep by her mother. As the light fades, the curtain drops, and on being almost immediately raised discloses a rose garden with the child Thomasina looking round in amazement. The toys have come to life, and, being introduced by "Alice in Wonderland," take part in an enjoyable programme of songs, quips, and dialogue. The second act shows Thomasina in the ante-room of a palace, where an "ancestor" in chain armour comes to life. Choruses were sung by pierrots, niggers, and "kitchen staff," the first and last singing topical songs about the surgical staff, the medical staff, the nursing staff, and the residents. All did excellently, but a special word of praise is due to the graceful dances by the Dancing Girl, the minuet by the Bear and the Fairy and the Ancestor and Thomasina, and to the by-play of the Bear and others and the splendid "fooling" of the Clown. It was altogether a grand show. All the expenses are covered by a fund raised by the house governor from members of the committee, staff, and friends, so that no cost falls on the hospital funds.

